# Delayed-Onset Neuropathic Pain after Septoplasty

**DOI:** 10.1155/2021/9966318

**Published:** 2021-12-22

**Authors:** Foteini-Stefania Koumpa, Mark Ferguson, Hesham Saleh

**Affiliations:** Imperial College NHS Trust, London, UK

## Abstract

Postoperative pain following a septoplasty is expected to be mild and limited to a few days after the operation. Chronic pain following the procedure is rare. No cases of delayed-onset neuropathic pain or allodynia have been described in the literature. This paper presents a case of delayed-onset neuropathic pain after septoplasty in a previously pain-free asthmatic patient that was successfully managed by administration of intranasal local anaesthesia. Physical examination and imaging excluded any other cause of neuralgia. A literature review revealed reports of chronic pain in patients following septoplasty if there were nasal contact or compression points or nasal tumours. Separately, acute postseptoplasty allodynia is documented in iatrogenic maxillary nerve damage. However, delayed-onset neuralgic pain, exacerbated by certain environmental triggers, has not been previously described. Facial pain can be debilitating; successfully managing this neuralgic pain with administration of intranasal local anaesthetic had a substantial effect on the patient's quality of life.

## 1. Introduction

Septoplasty is one of the most common operations performed by Ear, Nose, and Throat (ENT) surgeons internationally [1]. The operation involves correcting a deviated nasal septum to improve airflow bilaterally, and indications include a deviated septum causing nasal obstruction, allergic rhinitis, recurrent sinusitis, and epistaxis [2]. As with every surgical procedure, septoplasties have complications such as postoperative bleeding, septal haematoma, septal perforation, hyposmia, infection, adhesion formation, tooth anaesthesia, and rarer cranial or ocular complications [3]. In general, the postoperative pain associated with the procedure is reported as being mild to moderate and lasting 2 to 3 days and usually requires minimal use of opioid analgesics [4].

## 2. Case Report

A thirty-four-year-old radiographer presented four months after septoplasty with nasal blockage and irritability. She had previously presented to otolaryngology with nasal blockage symptoms, and although she was known to suffer with nonallergic rhinitis, her nasal obstruction was deemed to, in significant part, be due to the alignment of her septum. A septoplasty was undertaken to realign her septum with the aim of improving her nasal obstruction and facilitating the medical treatment of her nonallergic rhinitis (skin prick test was negative). She also had significant adenoidal hypertrophy, so additionally, an adenoidectomy was performed. Her past medical history included asthma, laryngeal hypersensitivity, nonallergic rhinitis, gastroesophageal reflux disease, long-standing vitamin D deficiency, and recurrent chest infections. She had no medical history of mood or psychiatric disorder. As a child, she had had a tonsillectomy. Her regular medications were prednisolone 10–15 mg OD which was required to control her asthma, omeprazole 40 mg BD, ranitidine 300 mg ON, Avamys spray OD, nasal douching regularly, evacal D3 tablets BD, salbutamol 100 microgram inhaler as needed, and tiotropium bromide 18 microgram inhaler OD. She has an allergy to penicillin.

A routine septoplasty was performed, but at routine postoperative review a few weeks later, the patient reported that she had a more irritable and runny nose than preoperatively whilst also feeling her nose was still blocked. On examination, she had a straight septum with inflamed mucosa without any evidence of nasal infection. The middle third of the septal cartilage had been resected with no mucosal defects or damage seen. As a result, she was initially managed with reassurance, Avamys spray, and sinus rinses. She continued experiencing nasal blockage with irritation, and she was given antihistamines and switched to Flixonase nasules. On further follow-up, one-year after septoplasty, the patient complained of a burning sensation on inspiration and on cold air exposure. Furthermore, she did not feel that Flixonase nasules had improved her symptoms. As a result, she was switched to fexofenadine tablets and started on low-dose amitriptyline in an attempt to treat any contributory neuropathic pain. Unfortunately, even with this regimen, the patient continued to experience nasal burning that overtime was intensifying. Glycerol nasal drops and regular sinus rinses were added to her treatment regime, and she gained some partial relief. Despite these interventions, the patient was still describing intense burning pain, particularly on inhalation of smoke, bleach, or cold air. Three years after her operation, the patient started describing the feeling of nasal streaming in addition to the burning sensation, despite her nose being dry on examination. The patient whenever questioned about the start of her nasal pain always linked it to the septoplasty.

On reexamination, there was no erythema, swelling, or deviation of her nose externally. Nasal endoscopy revealed that her septum was straight, there were no perforations, masses, or adhesions, and her nasal mucosa appeared healthy throughout.

**Figure 1:**
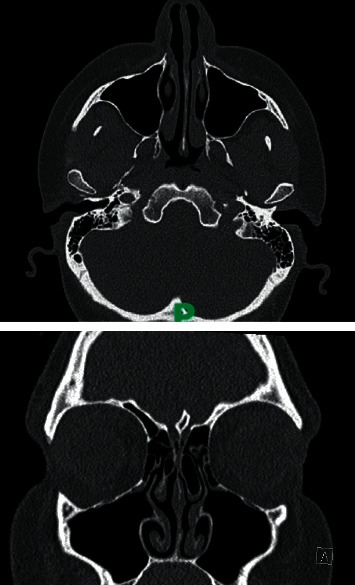
Computerised tomography scan of her sinuses indicating no anatomical causes.

Her inflammatory markers were within normal limits, and her skin prick test was normal. Her postoperative computerized tomography (CT) of her sinuses (Figure 1) did not show any abnormalities within the sinuses or skull base or any contact points.

Finally, a presumptive diagnosis of a postsurgical neuralgia triggered by specific stimuli was made, and the patient was trialed on intranasal local anaesthesia (lidocaine hydrochloride 5%w/v and phenylephrine hydrochloride 0.5%w/v). She was advised to apply 1 spray of the solution in each nostril when she knew she would be exposed to her specific triggers. She found this treatment strategy transformative, and she has managed to successfully control her nasal burning symptoms for the past 12 months.

This case is the second reported case of neuralgic nasal pain after septoplasty, but the first one reported with delayed onset. This neuralgic pain overtime was exacerbated by specific environmental triggers which before septoplasty caused no pain whatsoever. This debilitating pain was completely controlled with prophylactic administration of local anaesthetic which proved to be transformative for the patient on her last follow-up (4 years after surgery). We suggest that the pain was likely to be secondary to after surgical trigeminal nerve chemoreceptor dysregulation, but clearly, more research is needed to understand this interesting phenomenon.

## 3. Discussion

Nasal pain after septoplasty and septorhinoplasty is expected acutely, and numerous studies have been conducted looking into the optimum analgesia to manage short-term pain [4–6]. However, the length of postoperative pain is usually a few days rather than months or years. Generally, the pain is described as achy or throbbing rather than burning [4]. Furthermore, postseptoplasty sensory neuropathy has been reported following nerve trauma [7], but allodynia and chronic pain after septoplasty are not commonly seen. A systematic literature review was performed using the EMBASE and PUBMED databases from 1950 until October 2021. The key words used were the following: “septoplasty,” “rhinoplasty,” “turbinate surgery,” “septorhinoplasty,” and “nasal surgery” together with “pain” and “chronic pain.” The literature was reviewed according to the Preferred Reporting Items for Systematic Reviews and Meta-Analyses (PRISMA) framework. One similar case of postseptoplasty allodynia and four cases of postoperative nasal pain were identified. The cases of long-lasting nasal pain were secondary to contact points, implant displacement, suture-related neuronal compression, or neuroma formation.

Sharma et al. describe a case of maxillary nerve (V2) allodynia that developed following a septoplasty and deteriorated further following surgical revision [8]. The patient in their case study described hypersensitivity to temperature and gentle touch that was not relieved by topical or oral agents. Symptoms were firstly managed by infrazygomatic bilateral maxillary nerve (V2) blocks and subsequently with bilateral radiofrequency ablation of the maxillary branch of the trigeminal nerve.

Four cases of postoperative chronic pain following septoplasty were identified in the literature. In all four cases, a clear cause for the pain was found whether that was secondary to a contact point (Kaida et al. [9]), displacement of the nasal implant causing cutaneous nerve compression (Tseng and Chiu [10]), nasal suture placement that led to nerve compression (Swiss et al. [11]), or postoperative neuroma formation (Akbas et al. [12]).

In the cases mentioned above, the patients described unprovoked nasal pain that presented acutely following the surgery. In the case presented here, the patient experienced pain provoked by airflow and cold temperatures that developed 1 year following the septoplasty.

Nasal irritation is perceived when the trigeminal nerve chemoreceptors are stimulated at high concentrations of volatile organic compounds. This irritation is perceived as nasal itching, burning, and pain [13]. Allodynia is the perception of pain by a stimulus that usually does not cause pain such as cold temperatures or light touch [14]. Allodynia and hyperalgesia are known to result following nerve injury and are part of the neuropathic pain syndromes [14]. The mechanism behind neuropathic pain occurrence and maintenance is complicated but mainly involves the combination of peripheral and central sensitization. Peripheral nerve damage leads to inflammatory mediators that alter the properties of primary afferent neurons [15]. Peripheral sensitization reduces the threshold for neuronal firing via local changes in sodium, calcium, and potassium channels [16]. This leads to excitability, stimulus-independent activity, and spontaneous firing. The peripheral injury also causes central sensitization via influencing the plasticity of sensory processing in the spinal cord and higher centres. Lowering of activation thresholds, increase in receptor field size, and alteration of firing temporal dynamics can lead to a subthreshold input in the injured nerve to create a novel output [15, 16]. After injury, nerve fibres can undergo phenotypic change that maintains and triggers central sensitization [16]. This central plasticity can lead to neuropathic pain presence even after the local inflammation has resolved. In this case, we postulate that trauma to the distal nerve endings of the maxillary division of the trigeminal nerve during the septoplasty led to delayed-onset posttraumatic allodynia. As in this case, studies have shown that blocking the peripheral input with local anaesthesia can make the central processes revert to normal and eliminate symptoms during the effect of the anaesthesia [17]. The delay in the onset of allodynia is postulated to be due to the time taken for nerve sprouting and central sensitization to develop as well as the use of topical corticosteroid agents controlling the inflammation at a local level.

Trigeminal nerve injury is managed by surgery, local anaesthesia, and CBT [18]. In this case, the simple application of cophenylcaine spray prior to exposures that would lead to allodynia managed to successfully resolve the patient's symptoms. Thus, more invasive surgical options such as trigeminal ganglion ablation, stereotactic radiosurgery, or rhizotomy were not required.

## Data Availability

No further data were used to support this study.

## Abbreviations

OD: Once a day
BD: Twice a day
TDS: Three times a day
ENT: Ear, nose, and throat
CT: Computerised tomography
PRISMA: Preferred Reporting Items for Systematic Reviews and Meta-Analyses.
